# Missense Mutation in Exon 2 of SLC36A1 Responsible for Champagne Dilution in Horses

**DOI:** 10.1371/journal.pgen.1000195

**Published:** 2008-09-19

**Authors:** Deborah Cook, Samantha Brooks, Rebecca Bellone, Ernest Bailey

**Affiliations:** 1 MH Gluck Equine Research Center, Department of Veterinary Science, University of Kentucky, Lexington, Kentucky, United States of America; 2 Department of Animal Science, Cornell University, Ithaca, New York, United States of America; 3 Department of Biology, University of Tampa, Tampa, Florida, United States of America; Stanford University School of Medicine, United States of America

## Abstract

Champagne coat color in horses is controlled by a single, autosomal-dominant gene (*CH*). The phenotype produced by this gene is valued by many horse breeders, but can be difficult to distinguish from the effect produced by the *Cream* coat color dilution gene (*CR*). Three sires and their families segregating for *CH* were tested by genome scanning with microsatellite markers. The *CH* gene was mapped within a 6 cM region on horse chromosome 14 (LOD = 11.74 for θ = 0.00). Four candidate genes were identified within the region, namely *SPARC* [*Secreted protein*, *acidic*, *cysteine-rich (osteonectin)*], *SLC36A1* (*Solute Carrier 36 family A1*), *SLC36A2 (Solute Carrier 36 family A2)*, and *SLC36A3 (Solute Carrier 36 family A3)*. *SLC36A3* was not expressed in skin tissue and therefore not considered further. The other three genes were sequenced in homozygotes for *CH* and homozygotes for the absence of the dilution allele (*ch*). *SLC36A1* had a nucleotide substitution in exon 2 for horses with the champagne phenotype, which resulted in a transition from a threonine amino acid to an arginine amino acid (T63R). The association of the single nucleotide polymorphism (SNP) with the champagne dilution phenotype was complete, as determined by the presence of the nucleotide variant among all 85 horses with the champagne dilution phenotype and its absence among all 97 horses without the champagne phenotype. This is the first description of a phenotype associated with the *SLC36A1* gene.

## Author Summary

The purpose of this study was to uncover the molecular basis for the champagne hair color dilution phenotype in horses. Here, we report a DNA base substitution in the second exon of the horse gene *SLC36A1* that changes an amino acid in the transmembrane domain of the protein from threonine to arginine. The phenotypic effect of this base change is a diminution of hair and skin color intensity for both red and black pigment in horses, and the resulting dilution has become known as champagne. This is the first genetic variant reported for *SLC36A1* and the first evidence for its effect on eye, skin, and hair pigmentation. So far, no other phenotypic effects have been attributed to this gene. This discovery of the base substitution provides a molecular test for horse breeders to test their animals for the *Champagne* gene (*CH*).

## Introduction

Many horse breeders value animals with variation in coat color. Several genes are known which diminish the intensity of the coloration and are phenotypically described as “dilutions”. Two of these are a result of the *Cream (CR)* locus and *Silver* (*Z*) locus. The molecular basis for *Cream* is the result of a single base change in exon 2 of the *SLC45A2 (Solute Carrier 45 family A2*, *aka MATP* for *membrane associated transport protein*) on ECA21 [1],[3]. This change results in the replacement of a polar acidic aspartate with a polar neutral asparagine in a putative transmembrane region of the protein coded for by this gene [3],[2]. *CR* has an incompletely dominant mode of expression. Heterozygosity for *CR* dilutes only pheomelanin (red pigment) whereas homozygosity for *CR* results in extreme dilution of both pheomelanin and eumelanin (black pigment) [4].

The Silver dilution is the result of a missense mutation of *PMEL17* (Premelanosomal Protein) on ECA6. The base change causes replacement of a cytosolic polar neutral arginine with non-polar neutral cysteine in *PMEL17* [2]. In contrast to *CR*, the *Z* locus is fully dominant and affects only eumelanin causing little to no visible change in the amount of pheomelanin regardless of zygosity. The change in eumelanin is most apparent in the mane and tail where the black base color is diluted to white and gray [5].

**Figure 1:**
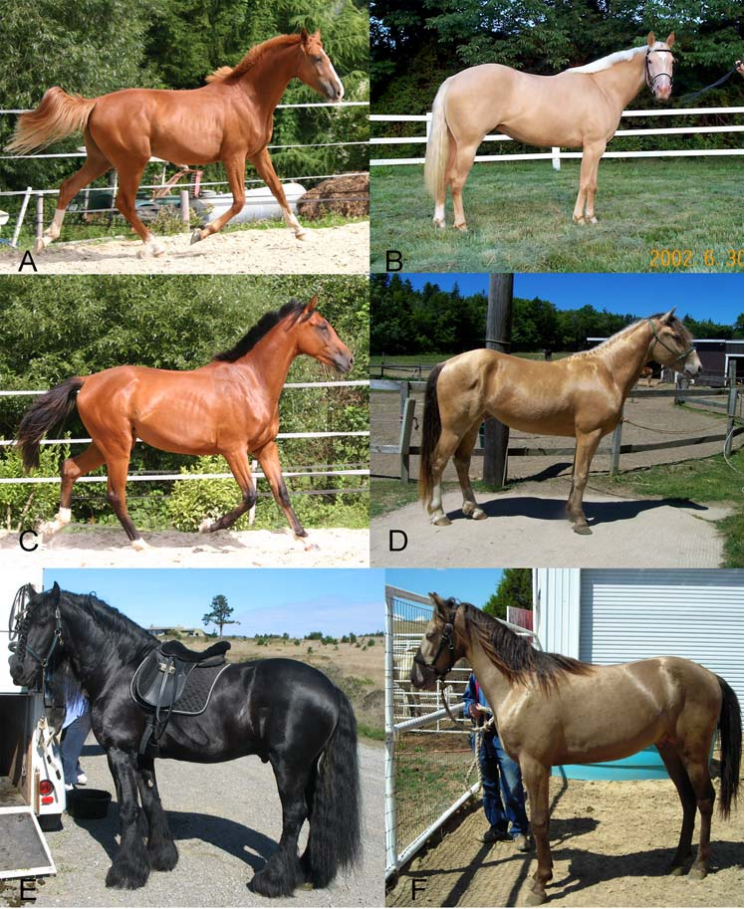
Effect of *Champagne* gene action on base coat colors of horses (chestnut, bay, and black). A) Chestnut – horse only produces red pigment. B) Chestnut diluted by *Champagne* = Gold Champagne. C) Bay – black pigment is limited to the points (e.g. mane, tail, and legs) allowing red pigment produced on the body to show. D) Bay diluted by *Champagne* = Amber Champagne. E) Black – red and black pigment produced, red masked by black. F) Black diluted by *Champagne* = Classic Champagne.

**Figure 2:**
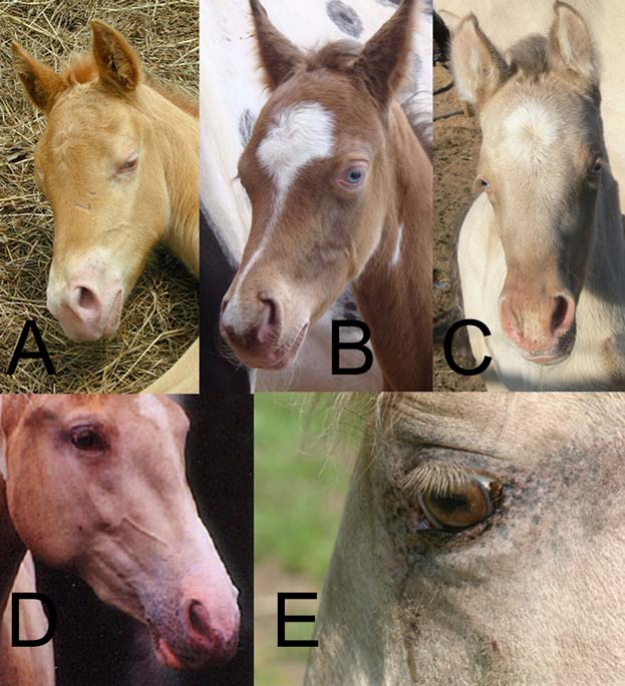
Champagne Eye and Skin traits. A, B and C) Eye and skin color of foals. D and E) Eye color and skin mottling of adult horse.

The coat color produced by the *CH* locus is similar to that of the *CR* locus in that both can cause dilution phenotypes affecting pheomelanin and eumelanin. However, the effect of *CH* differs from *CR* in that; 1) *CH* dilutes both pheomelanin and eumelanin in its heterozygous form and 2) heterozygotes and homozygotes for *CH* are phenotypically difficult to distinguish. The homozygote may differ by having less mottling or a slightly lighter hair color than the heterozygote. Figure 1 displays images of horses with the three base coat colors chestnut, bay and black and the effect of *CH* upon each. Figure 2 shows that champagne foals are born with blue eyes, which change color to amber, green, or light brown and pink “pumpkin skin which acquires a darker mottled complexion around the eyes, muzzle, and genitalia as the animal matures [6]. Foals with one copy of *CR* also have pink skin at birth but their skin is slightly darker and becomes black/near black with age. The champagne phenotype is found among horses of several breeds, including Tennessee Walking Horses and Quarter Horses. Here we describe family studies that led to mapping the gene and subsequent investigations leading to the identification of a genetic variant that appears to be responsible for the champagne dilution phenotype.

## Results

### Linkage Analyses

Table 1 summarizes the evidence for linkage of the *CH* gene to a region of ECA14. The linkage phase for each family was apparent based on the number of informative offspring in each family. Recombination rates (θ) were based on the combined recombination rate from all families. Four microsatellites showed significant linkage to the *CH* locus: *VHL209* (LOD = 6.03 for θ = 0.14), *TKY329* (LOD = 3.64 for θ = 0.10), *UM010* (LOD = 5.41 for θ = 0.04) and *COOK007* (LOD = 11.74 for θ = 0.00).

**Table 1:** Linkage Analysis between the Champagne Dilution and Microsatellite Markers; *UM010*, *COOK007*, *TKY329* and *VHL209*.

			alleles	Sire contribution					Statistics	
Sire Family	*(CH)*	microsatellite	*a/b*	N	a+	a−	b+	b−	LOD score	Θ
3	*(+/−)*	*UM010*	*124/108*	23	12	0	0	11	5.41	
								**Σ = **	**5.41**	0
1	*(+/−)*	*COOK007*	*332/334*	14	10	0	0	4	4.21	
2	*(+/−)*	*COOK007*	*332/334*	8	4	0	0	4	2.41	
3	*(+/−)*	*COOK007*	*332/324*	17	8	0	0	9	5.12	
								**Σ = **	**11.74**	0
1	*(+/−)*	*TKY329*	*117/139*	15	10	2	0	3	1.92	
2	*(+/−)*	*TKY329*	*111/137*	9	5	1	0	3	1.34	
3	*(+/−)*	*TKY329*	*117/139*	18	7	0	1	10	3.64	
								**Σ = **	**6.9**	0.1
1	*(+/−)*	*VHL209*	*95/93*	13	4	1	1	7	1.49	
2	*(+/−)*	*VHL209*	*91/93*	12	4	2	1	5	0.46	
3	*(+/−)*	*VHL209*	*95/93*	24	10	1	1	12	4.08	
								**Σ = **	**6.03**	0.14

N = the number of informative meiosis.

Θ = recombination frequency between that microsatelite and the *champagne gene* for all families combined.

Σ = LOD score for which 1/10^Σ^ = the odds the association between the phenotype and the marker is due to chance.

**Figure 3:**
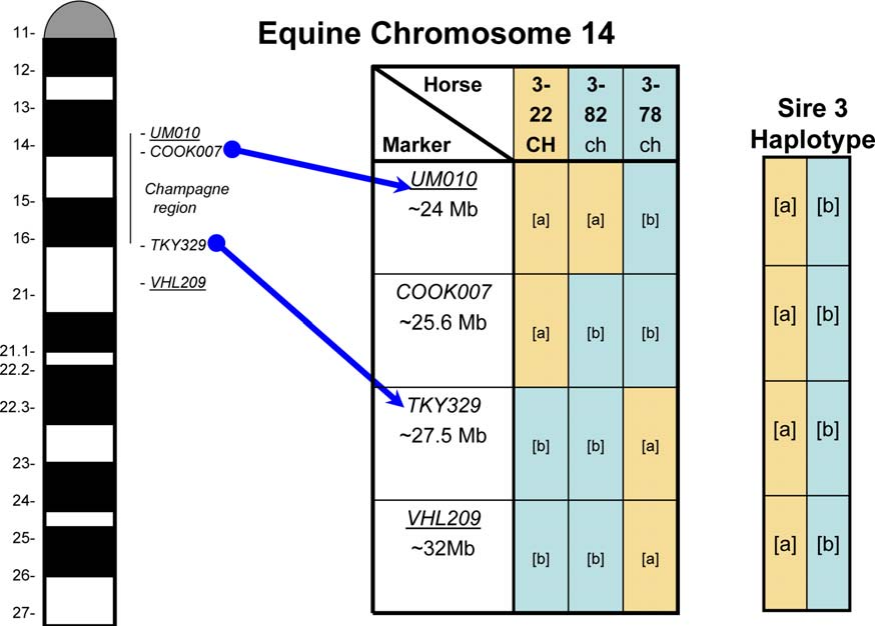
Example of Recombinant Haplotypes. Linear relationship from top to bottom between the microsatellites, phenotype, and genotype of recombinant offspring for study sire #3. Phenotype is noted in top row with offspring's ID #.

Figure 3 identifies the haplotypes for offspring of a single sire showing recombination between the genetic markers and the *CH* locus. Pedigrees of the three sire families and haplotype information are provided in Figure S1 and Table S1 respectively. The *CH* locus maps to an interval between *UM010* and *TKY329* with microsatellite. No recombinants were detected among 39 informative offspring between the *CH* and *COOK007* locus.

### Candidate Genes

Candidate genes were selected on the basis of proximity to the marker *COOK007* and as genes previously characterized in other species as influential in the production or migration of pigment cells.

*SPARC* was located closest at ∼90 kb downstream from *COOK007* and is coded for on the plus strand of DNA. It has been implicated in migration of retinal pigment epithelial cells in mice [7].

*SLC36A* family members are solute carriers and other solute carrier families have been found to play a role in coat color. *SLC36A1* is located ∼250 kb downstream from *COOK007*. It is the first and most proximal to *COOK007* of three genes in this family and is coded for on the minus strand of DNA.

*SLC36A2* and *SLC36A3* are coded for on the plus strand of DNA and are approximately 350 k and 380 k downstream from *COOK007* respectively. *A2* and *A3* have been found to be expressed in a limited range of tissues in humans and mice [8].

### RT-PCR

**Figure 4:**
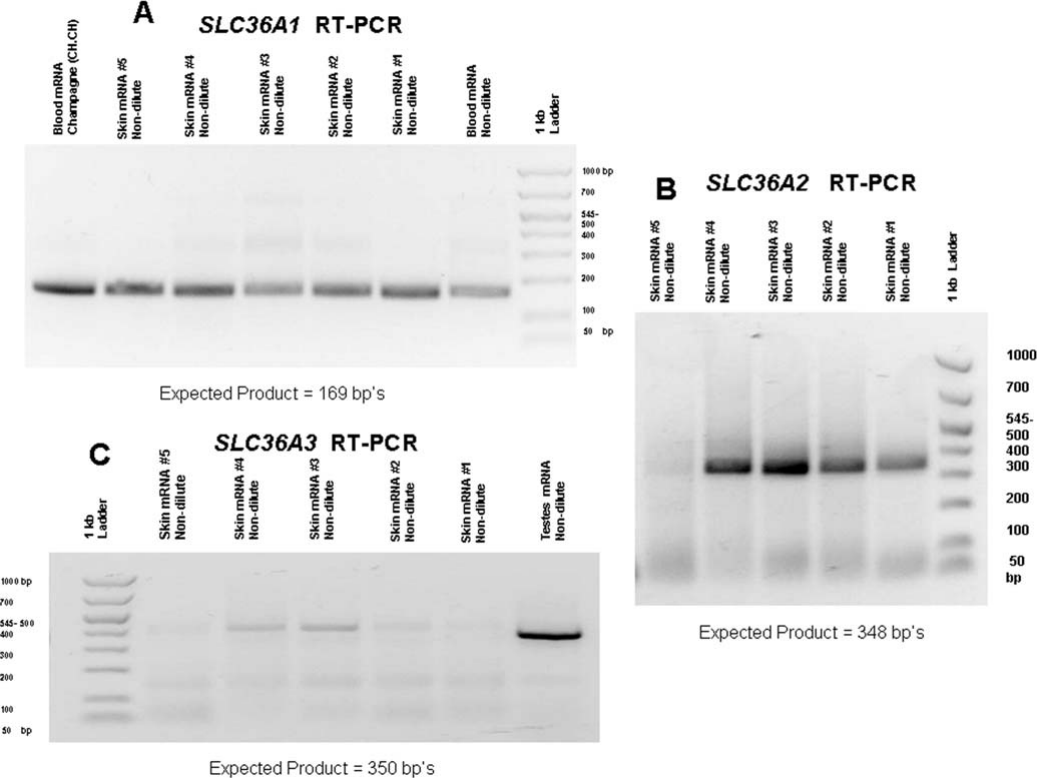
RT-PCR product results for *SLC36A1*, *A2* and *A3*. A) RT-PCR results for *SLC36A1*. B) RT-PCR results for *SLC36A2*. C) RT-PCR results for *SLC36A3*. (Faint bands observed above 400 bp on gel C were sequenced and did not show homology to *SLC36A3*.)

RT-PCR (reverse transcription-polymerase chain reaction) was used to determine if *SLC36A1*, *SLC36A2* or *SLC36A3* were expressed in skin. *SLC36A1* and *SLC36A2* were expressed in skin and their genomic exons were sequenced. *SLC36A3* was not detected in skin and therefore not investigated for detection of SNPs. Results for RT-PCR of these three genes are shown in Figure 4.

### Sequencing

All 9 exons of *SPARC* were sequenced. Three SNPs were found in exons but none showed associations with the champagne phenotype and are shown in Table S2.

*SLC36A2* was sequenced with discovery of 9 SNPs in exons. None of the SNPs showed associations with CH. These SNPs and all other variations detected are described in Table S2.

**Figure 5:**
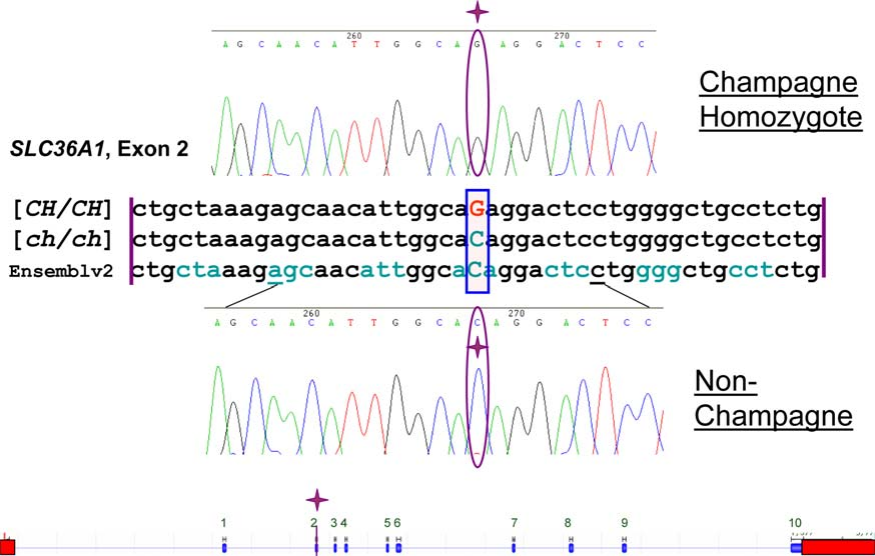
Sequence Alignment and Gene Diagram. Alignment is between homozygous champagne, non-dilute, and horse genome assembly. Reading frame is marked by alternating colors of codons. Bottom is diagram of *SLC36A1* with the identified SNP in exon 2. Sequence and gene layout have been verified on Ensembl genome browser equine assembly v2. Blue blocks of gene layout are exons and red boxes are the 5′ and 3′ UTRs.

*SLC36A1* was sequenced. Only one SNP was found, a missense mutation involving a single nucleotide change from a C to a G at base 76 of exon 2 (c.188C>G) (Figure 5). These *SLC36A1* alleles were designated *c.188[C/G]*, where c.188 designates the base pair location of the SNP from the first base of *SLC36A1* cDNA, exon 1. Sequencing traces for the partial coding sequence of *SLC36A1* exon 2 with part of the flanking intronic regions for one non-champagne horse and one champagne horse were deposited in GenBank with the following accession numbers respectively: EU432176 and EU432177. This single base change at *c.188* was predicted to cause a transition from a threonine to arginine at amino acid 63 of the protein (T63R).

### Protein Alignment

**Figure 6:**
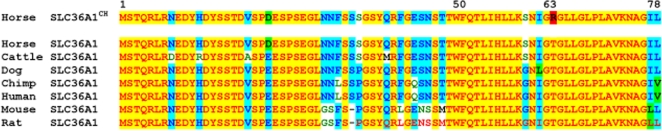
Seven Species Protein Sequence Alignment for *SLC36A1* exons 1 and 2. The R highlighted in red is the amino acid replacement associated with the champagne phenotype.

Figure 6 shows the alignment of the protein sequence for exons 1 and 2 of *SLC36A1* for seven mammalian species with sequence information from Genbank (horse, cattle, chimpanzee, human, dog, rat and mouse). Alignment was performed using AllignX function of Vector NTI Advance 10 (Invitrogen Corp, Carlsbad, California). The alignment demonstrates that this region is highly conserved among all species. At position 63, the amino acid sequence is completely conserved among these species, with the exception of horses possessing the champagne phenotype. This replacement of threonine with arginine occurs in a putative transmembrane domain of the protein [9].

### Population Data

The distribution of *c.188G* allele among different horse breeds and among horses with and without the champagne phenotype was investigated. Table 2 is a compilation of the population data collected via the genotyping assay. All dilute horses (85) which did not have the *CR* gene, tested positive for the *c.188G* allele with genotypes *c.188C/G* or *c.188G/G*. No horses in the non-dilute control group (97) possessed the *c.188G* allele. The horses used for the population study were selected for coat color and not by random selection; therefore measures of Hardy-Weinberg equilibrium are not applicable and were not calculated.

**Table 2:** Genotyping Results for c.188(C/G) locus.

	*Champagne (CH/CH or CH/ch)*		*Non-Dilute (ch/ch, cr/cr)*	
Horse Breeds	*G/G*	*G/C*	*C/C*	Total
American Cream Draft Cross	0	1	0	1
American Miniature Horse	0	9	1	10
American Quarter Horse	1	26	1	28
American Paint Horse	0	13	32	45
American Saddlebred	0	2	2	4
Appaloosa	0	1	0	1
Kentucky Mountain	0	1	0	1
Part Arabian	0	3	0	3
Pinto	0	0	5	5
Pony	0	1	1	2
Missouri Foxtrotter	0	4	0	4
Mule	0	2	0	2
Spanish Mustang	0	1	0	1
Spotted Saddle Horse	0	1	0	1
Tennessee Walking Horse	3	17	20	40
Thoroughbred	0	0	35	35
**Total**	4	82	97	183

## Discussion

Family studies clearly showed linkage of the gene for the champagne dilution phenotype within a 6 cM region on ECA14 [10] (Table 1). Based on the Equine Genome Assembly V2 as viewed in ENSEMBL genome browser (http://www.ensembl.org/Equus_caballus/index.html) this region spans approximately 2.86 Mbp [11]. Within that region, four candidate genes were investigated; one based on known effects on melanocytes (eg. *SPARC*) and three for their similarity to other genes previously shown to influence pigmentation (eg, *SLC36A1*, *A2*, *and A3*). While SNPs were found within the exons of SPARC, none were associated with *CH*. Of the other 3 candidate genes, only *SLC36A1* and *SLC36A2* were found to be expressed in skin cells. Therefore, the exons of those two genes were sequenced. A missense mutation in the second exon of *SLC36A1* showed complete association with the champagne phenotype across several breeds. While SNPs were found for *SLC36A2*, none showed associations at the population level for the champagne dilution phenotype.

This observation is the first demonstration for a role of *SLC36A1* in pigmentation. Orthologous genes in other species are known to affect pigmentation. For example, the gene responsible for the cream dilution phenotypes in horses, *SLC45A2 (MATP)*, belongs to a similar solute carrier family. In humans, variants in *SLC45A2* have been associated with skin color variation [12] and a similar missense mutation (p.Ala111Thr) in *SLC24A5* (a member of potassium-dependent sodium-calcium exchanger family) is implicated in dilute skin colors caused from decreased melanin content among people of European ancestry [13]. The same gene, *SLC24A5* is responsible for the *Golden (gol)* dilution as mentioned in the review of mouse pigment research by Hoekstra (2006) [14]

It is proposed, here, that the missense mutation in exon 2 of *SLC36A1* is the molecular basis for champagne dilution phenotype. While this study provides evidence that this is the mutation responsible for the champagne phenotype, the proof is of a statistical nature and a non-coding causative mutation can not be ruled out at this point. *SLC36A1*, previously referred to by the name *PAT1* (proton/amino acid transporter 1) in human and mouse [15], is a proton coupled small amino acid transporter located and most active in the brush border membranes of intestinal epithelial cells. This protein has also been characterized in rats under the name *LYAAT1* (*lysosomal amino acid transporter 1*). *LYAAT1* is localized in the membrane of lysozomes in association with LAMP1 (lysosomal associated protein 1) and in the cell membrane of post-synaptic junctions. In lysozomes it allows outward transport of protons and amino acids from the lysozome to the cytosol [16]. During purification and separation of early-stage melanosomes *LAMP1* is found in high concentrations in the fraction containing stage II melanosomes [17],. Perhaps *SLC36A1* plays a role in transitions from lysozome-like precursor to melanosome. Since organellular pH affects tyrosine processing and sorting [18], an amino acid substitution in this protein may affect pH of the early stage melanosome and the ability to process tyrosine properly. There must be an increase in pH, before the tyrosinase can be activated. The cytosolic pH gradient must also be maintained for proper sorting and delivery of the other proteins required for melanosome development [19]. Thus, the pH gradient of the cell may be altered by this mutation.

This variant, discovered in association with a coat dilution in the horse, is the first reported for the *SLC36A1* gene. The phenotype resulting from this mutation, a reduction of pigmentation in the eyes, skin and hair, illustrates previously unknown functions of the protein product of *SLC36A1*. Furthermore, now that a molecular test for champagne dilution is established, the genotyping assay can be used in concert with available tests for cream dilution and silver dilution to clarify the genetic basis of a horse's dilution phenotype. This will give breeders a new tool to use in developing their breeding programs whether they desire to breed for these dilutions or to select against them.

## Materials and Methods

### Horses

Three half-sibling families, designated 1, 2 and 3, were used for mapping studies. Family 1 consisted of a Tennessee Walking Horse (TWH) stallion, known heterozygous at the *Champagne locus* (*CH/ch*), and his 17 offspring out of non-dilute mares (ch/ch). Family 2 consisted of an American Paint Horse stallion (*CH/ch*) and his 10 offspring out of non-dilute (ch/ch) mares. Family 3 consisted of a TWH stallion (*CH/ch*), 23 offspring and their 12 non-dilute dams (ch/ch) and 1 dilute (buckskin) dam (ch/ch, *CR/cr*).

To investigate the distribution of the gene among dilute and non-dilute horses of different horse breeds, 97 non-champagne horses were chosen from stocks previously collected and archived at the MH Gluck Equine Research Center. These horses were from the following breeds: TWH (20), Thoroughbreds (TB, 35), American Paint Horses (APHA, 32), Pintos (5), American Saddlebreds (ASB, 2), one American Quarter Horse (AQHA), one pony, and one American Miniature (AMH) Horse.

Hair and blood samples from horses with the champagne dilution phenotype were submitted by owners along with pedigree information and photographs showing the champagne color and characteristics of each horse. Samples were collected from the following breeds (85 total): American Miniature Horse (9), American Cream Draft cross (1), American Quarter Horse (27), American Paint Horse (13, in addition to the family), American Saddlebred (2), Appaloosa (1), ASB/Friesian cross (1), Arabian crossed with APHA or AQHA horses (3), Missouri Foxtrotter(4), Mule (2), Pony (1), Spanish Mustang 1), Spotted Saddle Horse (1), Tennessee Walking Horse (20, in addition to the families).

### Color Determination

To be characterized as possessing the champagne phenotype, horses exhibited a diminished intensity of color (dilution) in black or brown hair pigment and met at least two of the three following criteria: 1) mottled skin around eyes, muzzle and/or genitalia, 2) amber, green, or light brown eyes, or 3) blue eyes and pink skin at birth [6]. This was accomplished by viewing photo evidence of these traits or by personal inspection. Due to potential confusion between phenotypes of cream dilution and champagne dilution, all DNA samples from horses with the dilute phenotype were tested for the *CR* allele and data from those testing positive were not included in the population data.

### DNA Extraction

DNA from blood samples was extracted using Puregene whole blood extraction kit (Gentra Systems Inc., Minneapolis, MN) according to its published protocol. Hair samples submitted by owners were processed using 5–7 hair bulbs according to the method described by Locke et al. (2002). The hair bulbs were placed in 100 µl lysis solution of 1× FastStart Taq Polymerase PCR buffer (Roche, Mannheim, Germany), 2.5 mM MgCl_2_ (Roche), 0.5% Tween 20 (JT Baker, Phillipsburg, NJ) and 0.01 mg proteinase K (Sigma-Aldrich, St Louis, MO) and incubated at 60°C for 45 minutes, followed by 95°C for 45 min to deactivate the proteinase K.

### Microsatellite Genome Scan

The genome scan was done in polymerase chain reaction (PCR) multiplexes of 3 to 6 microsatellites per reaction. The 102 microsatellite markers used are listed in Table S3. Primers for these microsatellites were made available in connection with the USDA-NRSP8 project [20]. Two additional microsatellites were used; *TKY329* [21] was selected based on its map location between two microsatellites used for genome scanning (*UM010* and *VHL209*) and *COOK007* was developed in connection with this study based on DNA sequence information from the horse genome sequence viewed in the UCSC genome browser [8] in order to investigate linkage within the identified interval. Primers for *COOK007* were designed using Primer 3 software accessed online (Forward, 5′- 6FAM-CATTCCAAACACCAACAACC - 3′), (Reverse, 5′ – GGACATTCCAGCAATACAGAG – 3′) [22]. The initial scan was conducted on a subset of samples from Family 3; including sire 3, five non-champagne offspring and five champagne offspring. When the microsatellite allele contribution from the sire was not informative, (e.g. the sire and offspring had the same genotype), dams from family 3 were typed to determine the precise contribution from the sire. When the inheritance of microsatellite markers in family 3 appeared to be correlated with the inheritance of the *CH* allele, then the complete families A, B and C were typed and the data analyzed for linkage by LOD score analysis [23].

Amplification for fragment analysis was done in 10 µl PCR reactions using 1× PCR buffer with 2.0 mM MgCl_2_, 200 µM of each dNTP, 1 µl genomic DNA from hair lysate, 0.1 U FastStart Taq DNA polymerase (Perkin Elmer, Waltham, MA) and the individual required molarity of each primer from the fluorescently labeled microsatellite parentage panel primer stocks at the MH Gluck Equine Research Center. Samples were run on a PTC-200 thermocycler (MJ research, Inc., Boston, MA) at a previously determined optimum annealing temperature for each multiplex. Capillary electrophoresis of product was run on an ABI 310 genetic analyzer (Applied Biosystems Inc. ABI, Foster City, CA). Results were then analyzed using the current version of STRand microsatellite analysis software (http://www.vgl.ucdavis.edu/informatics/STRand/).

### Sequencing

PCR template for sequencing was amplified in 20 µl PCR reactions using 1× PCR buffer with 2.0 mM MgCl_2_, 200 µM of each dNTP, 1 µl genomic DNA from hair lysate, 0.2 U FastStart Taq DNA polymerase (Perkin Elmer) and 50 nM of each primer. Exon 2 of *SLC36A1* was sequenced with the following primers: Forward (5′-CAG AGC CTA AGC CCA GTG TC-3′) and Reverse (5′-GGA GGA CTG TGT GGA AAT GG-3′) at an annealing temperature of 57°C. Primers used to sequence the other *SLC36A1* exons and primers for sequencing genomic exons of SLC36A2 are provided in parts 1 and 2 respectively of Table S4. Template product was quantified on a 1% agarose gel, then amplified with BigDye Terminator v1.1 cycle sequencing kit according to manufacturer's instructions (Applied Biosystems), cleaned using Centri-Sep columns (Princeton Separations Inc., Adelphia, NJ), and run on and ABI 310 genetic analyzer (Applied Biosystems). Six samples were initially sequenced: 2 suspected homozygous champagnes (based on production of all champagne dilution offspring when bred to at least 10 non-dilute dams), 2 heterozygotes, and 2 non-dilute horses. The results were analyzed and compared by alignment using ContigExpress from the Vector NTI Advance 10.3 software package (Invitrogen Corporation, Carlsbad, California).

### Reverse Transcription (RT-PCR)

RT-PCR was performed in 25 µl reactions a Titan One Tube RT-PCR Kit (Roche) according to enclosed protocol with the primers listed in part 3 of Table S4. RNA from different tissues of non-dilute horses was used to acquire partial cDNAs containing the first two exons for *SLC36A1*, first three exons *SLC36A2* and first 4 exons of *SLC36A3*. The cDNA acquired was sequenced and the resulting sequences were verified for their respective genes with a BLAT search using the equine assembly v2 in ENSEMBL (http://www.ensembl.org/Equus_caballus/index.html) genome browser. RT-PCR was also performed utilizing RNA extracted from skin, kidney and testes of non-dilute animals currently in lab stocks. *SLC36A1* cDNA was produced from the skin and blood using 50 ng RNA per reaction. *SLC36A2* cDNA was produced from testes using 1 mRNA per RT-PCR reaction then following up with a nested PCR for shorter product. *SLC36A2* cDNA was produced from skin using 50 ng mRNA per RT-PCR reaction. Nested PCR was not necessary. *SLC36A3* cDNA was produced from testes using 1 ug mRNA per reaction. 9 µl of initial reaction was visualized on a 2% agarose gel to check for visible bands of product. When product was not initially detected an additional 20 µl PCR was performed in reactions as outlined above using 5 µl of RT product in the place of hair lysate per reaction. Detected product was then sequenced with the protocol listed above. Sequences were then used in a BLAST search using equine genome assembly 2 on ENSEMBL genome browser to verify the correct cDNA was amplified.

### Custom TaqMan Probe Assay

A Custom TaqMan SNP Genotyping Assay (Applied Biosystems) was designed for c.188C/G SNP in filebuilder 3.1 software (Applied Biosystems) to test the population distribution of the *SLC36A1* alleles. A similar assay was also designed to test for the cream SNP. These assays were run on a 7500HT Fast Real Time-PCR System (Applied Biosystems). All dilute horses tested for *SLC36A1* variants were concurrently tested for *SLC45A* variants. Horses testing positive for *CR* alleles were not used in the dataset to avoid any confusion over the origin of their dilution phenotype.

## Supporting Information

Figure S1 Pedigrees of Three Sire Families used in Genome Scan.
(0.55 MB TIF)

Table S1 Haplotype Data for Three Sire Families.
(0.25 MB DOC)

Table S2 Sequence Variants Detected in *SPARC*, *SLC36A1*, and *SLC36A2*.
(0.14 MB DOC)

Table S3 Microsatellite Markers used For Genome Scan.
(0.20 MB DOC)

Table S4 Sequencing and RT-PCR Primers.
(0.07 MB DOC)
