# Supplementary figures and images for: Missense Mutation in Exon 2 of SLC36A1 Responsible for Champagne Dilution in Horses
Source: PLoS Genet. 2008 Sep 19;4(9):e1000195. doi: 10.1371/journal.pgen.1000195 (PMC2535566; doi:10.1371/journal.pgen.1000195)

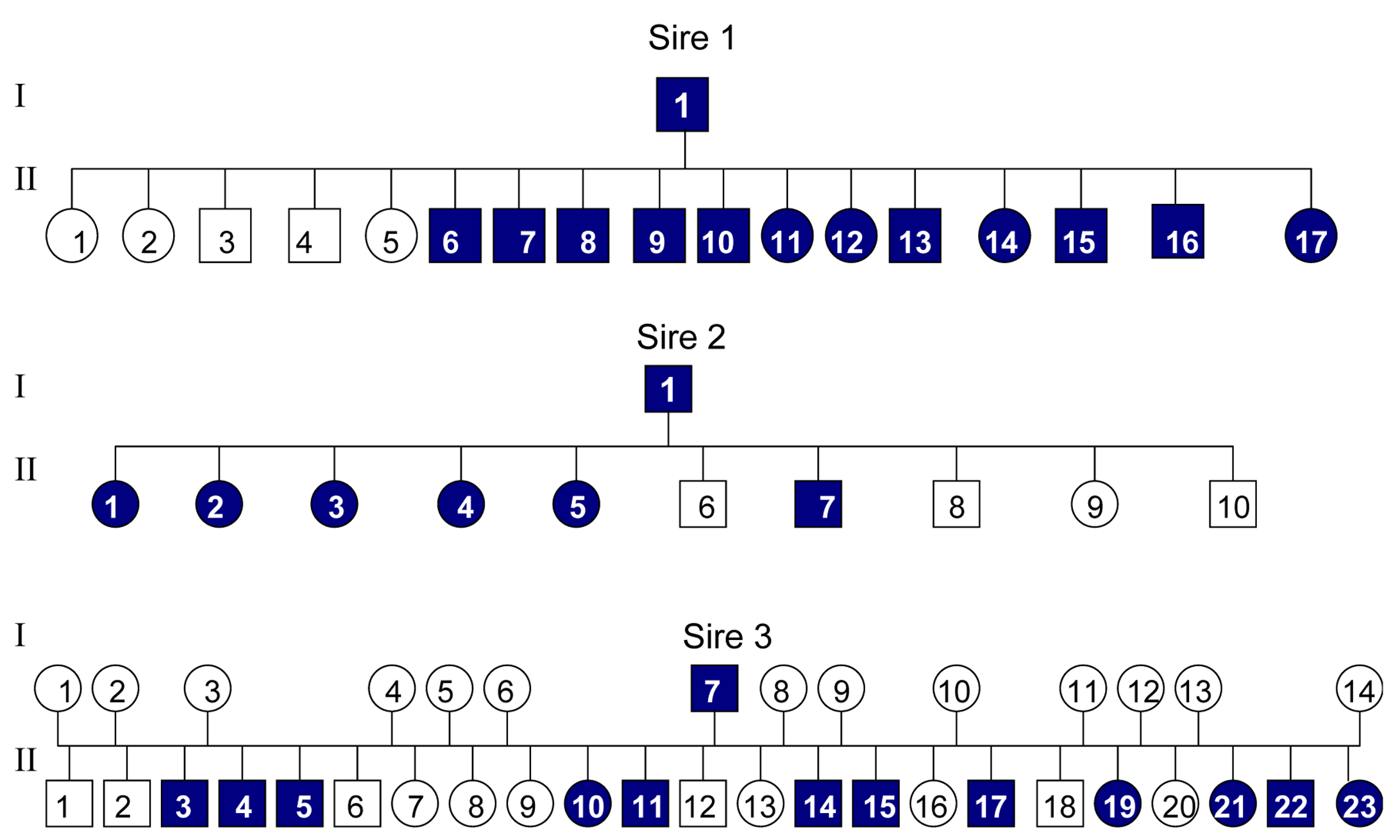

Supplement: Figure S1 — Pedigrees of Three Sire Families used in Genome Scan. (0.55 MB TIF) [file pgen.1000195.s001.tif]
