# Supplementary material for: Missense Mutation in Exon 2 of SLC36A1 Responsible for Champagne Dilution in Horses
Source: PLoS Genet. 2008 Sep 19;4(9):e1000195. doi: 10.1371/journal.pgen.1000195 (PMC2535566; doi:10.1371/journal.pgen.1000195)
Supplement: Table S1 — Haplotype Data for Three Sire Families. (0.25 MB DOC) [file pgen.1000195.s002.doc]

| **Haplotype Data for 3 Families**  **From Supplemental Figure 1 Pedigree Chart**  **SIRE 1** | | | | | | | | | | | | | |
| --- | --- | --- | --- | --- | --- | --- | --- | --- | --- | --- | --- | --- | --- |
| **Sire 1** | **Color** | **Sex** | **UM010** | |  | **COOK007** | |  | **TKY329** | |  | **VHl209** | |
| I-1 | CH | Sire | 124 | 124 |  | 332 | 334 |  | 117 | 139 |  | 93 | 95 |
| II-1 | ch | female | -- | -- |  | 334 | 338 |  | 117 | 127 |  | 87 | 93 |
| II-2 | ch | female | 108 | 124 |  | 324 | 334 |  | 139 | 139 |  | 93 | 95 |
| II-3 | ch | male | 116 | 124 |  | 332 | 334 |  | 117 | 127 |  | 85 | 95 |
| II-4 | ch | male | -- | -- |  | 334 | 338 |  | 127 | 139 |  | 87 | 93 |
| II-5 | ch | female | 122 | 124 |  | 334 | 338 |  | 137 | 139 |  | 85 | 93 |
| II-6 | CH | male | 122 | 124 |  | -- | -- |  | -- | -- |  | 93 | 95 |
| II-7 | CH | male | 110 | 124 |  | 332 | 342 |  | 117 | 123 |  | 93 | 95 |
| II-8 | CH | male | -- | -- |  | 332 | 332 |  | 117 | 137 |  | 95 | 95 |
| II-9 | CH | male | 122 | 124 |  | 332 | 338 |  | 117 | 137 |  | 85 | 95 |
| II-10 | CH | male | 122 | 124 |  | 332 | 338 |  | 117 | 137 |  | 85 | 95 |
| II-11 | CH | female | 116 | 124 |  | -- | -- |  | -- | -- |  | 93 | 95 |
| II-12 | CH | female | -- | -- |  | 332 | 338 |  | 117 | 135 |  | 93 | 95 |
| II-13 | CH | male | -- | -- |  | 332 | 338 |  | 117 | 137 |  | 87 | 95 |
| II-14 | CH | female | -- | -- |  | 332 | 332 |  | 117 | 137 |  | 87 | 95 |
| II-15 | CH | male | -- | -- |  | 324 | 332 |  | 117 | 135 |  | 87 | 95 |
| II-16 | CH | male | -- | -- |  | 332 | 338 |  | 117 | 127 |  | 87 | 93 |
| II-17 | CH | female | -- | -- |  | 332 | 338 |  | 117 | 127 |  | 87 | 95 |
| **SIRE 2** | | | | | | | | | | | | | |
| **Sire 2** | **Color** | **Sex** | **UM010** | |  | **COOK007** | |  | **TKY329** | |  | **VHl209** | |
| I-1 | CH | Sire | -- | -- |  | 332 | 334 |  | 111 | 137 |  | 91 | 93 |
| II-1 | CH | female | -- | -- |  | 332 | 338 |  | 111 | 139 |  | 87 | 93 |
| II-2 | CH | female | -- | -- |  | 332 | 332 |  | 111 | 139 |  | 91 | 101 |
| II-3 | CH | female | -- | -- |  | 332 | 332 |  | 111 | 139 |  | 91 | 93 |
| II-4 | CH | female | -- | -- |  | -- | -- |  | 111 | 139 |  | 87 | 91 |
| II-5 | CH | female | -- | -- |  | 332 | 334 |  | 117 | 137 |  | 87 | 91 |
| II-6 | ch | male | -- | -- |  | 334 | 338 |  | 111 | 137 |  | 93 | 95 |
| II-7 | CH | male | -- | -- |  | 330 | 332 |  | 111 | 139 |  | 87 | 91 |
| II-8 | ch | male | -- | -- |  | 334 | 338 |  | 137 | 139 |  | 93 | 95 |
| II-9 | ch | female | -- | -- |  | 324 | 334 |  | 137 | 139 |  | 87 | 93 |
| II-10 | ch | male | -- | -- |  | 332 | 334 |  | 111 | 127 |  | 87 | 91 |
| **SIRE 3** | | | | | | | | | | | | | |
| **Family 3** | **Color** | **Sex** | **UM010** | |  | **COOK007** | |  | **TKY329** | |  | **VHl209** | |
| I-7 | CH | Sire | 108 | 124 |  | 324 | 332 |  | 117 | 139 |  | 93 | 95 |
|  |  |  |  |  |  |  |  |  |  |  |  |  |  |
| I-1 | ch | Dam | 108 | 124 |  | 324 | 338 |  | 127 | 139 |  | 85 | 93 |
| II-1 | ch | male | 108 | 108 |  | 324 | 324 |  | 139 | 139 |  | 93 | 93 |
| I-2 | ch | Dam | 108 | 124 |  | 324 | 338 |  | 127 | 139 |  | 85 | 85 |
| II-2 | ch | male | 108 | 124 |  | 324 | 338 |  | 127 | 139 |  | 85 | 93 |
| II-3 | CH | male | 124 | 124 |  | 332 | 338 |  | 117 | 127 |  | 85 | 95 |
| I-3 | ch | Dam | 122 | 124 |  | 332 | 338 |  | 127 | 127 |  | 85 | 93 |
| II-4 | CH | male | 124 | 124 |  | 332 | 332 |  | 117 | 127 |  | 85 | 95 |
| II-5 | CH | male | 124 | 124 |  | 332 | 338 |  | 117 | 127 |  | 85 | 95 |
| II-6 | ch | male | 108 | 122 |  | 324 | 332 |  | 127 | 139 |  | 93 | 93 |
| I-4 | ch | Dam | 108 | 108 |  | 324 | 324 |  | 139 | 139 |  | 85 | 93 |
| II-7 | ch | female | 108 | 108 |  | 324 | 324 |  | 139 | 139 |  | 85 | 93 |
| I-5 | ch | Dam | 116 | 124 |  | 338 | 338 |  | 127 | 127 |  | 85 | 93 |
| II-8 | ch | female | 108 | 116 |  | 324 | 338 |  | 127 | 139 |  | 93 | 93 |
| I-6 | ch | Dam | 118 | 122 |  | 324 | 332 |  | 137 | 139 |  | 85 | 85 |
| II-9 | ch | female | 108 | 122 |  | 324 | 332 |  | 137 | 139 |  | 85 | 93 |
| II-10 | CH | female | 118 | 124 |  | 324 | 332 |  | 117 | 139 |  | 85 | 95 |
| II-11 | CH | male | 118 | 124 |  | 324 | 332 |  | 117 | 139 |  | 85 | 95 |
| II-12 | ch | male | 108 | 118 |  | 324 | 324 |  | 139 | 139 |  | 85 | 93 |
| I-8 | ch | Dam | -- | -- |  | -- | -- |  | -- | -- |  | -- | -- |
| II-13 | ch | female | 108 | 108 |  | 324 | 324 |  | 139 | 139 |  | 93 | 93 |
| I-9 | ch | Dam | 120 | 124 |  | 334 | 338 |  | 133 | 137 |  | 85 | 85 |
| II-14 | CH | male | 108 | 124 |  | 332 | 338 |  | 133 | 139 |  | 85 | 93 |
| I-10 | ch | Dam | 108 | 114 |  | 324 | 342 |  | 139 | 139 |  | 93 | 93 |
| II-15 | CH | male | 108 | 124 |  | 324 | 332 |  | 117 | 139 |  | 93 | 95 |
| II-16 | ch | female | 108 | 114 |  | 324 | 342 |  | 117 | 139 |  | 93 | 95 |
| I-11 | ch | Dam | 124 | 124 |  | 332 | 338 |  | 137 | 139 |  | 85 | 85 |
| II-17 | CH | male | 124 | 124 |  | 332 | 332 |  | 117 | 139 |  | 85 | 95 |
| II-18 | ch | male | 124 | 124 |  | 324 | 332 |  | 137 | 139 |  | 85 | 93 |
| I-12 | ch | Dam | 108 | 116 |  | 324 | 332 |  | 133 | 139 |  | 93 | 95 |
| II-19 | CH | female | 108 | 124 |  | 324 | 332 |  | 117 | 139 |  | 93 | 95 |
| I-13 | ch | Dam | 108 | 114 |  | 324 | 342 |  | 139 | 139 |  | 93 | 93 |
| II-20 | ch | female | 108 | 114 |  | 324 | 342 |  | 139 | 139 |  | 93 | 93 |
| I-14 | ch | Dam | 122 | 122 |  | 324 | 332 |  | 117 | 127 |  | 85 | 95 |
| II-21 | CH | female | 122 | 124 |  | 324 | 332 |  | 117 | 117 |  | 95 | 95 |
| II-22 | CH | male | 122 | 124 |  | 332 | 332 |  | 117 | 127 |  | 85 | 95 |
| II-23 | CH | female | 122 | 124 |  | 332 | 332 |  | 117 | 127 |  | 85 | 95 |

Supplemental Table 1:

Sire is the first row in each sub-table.

Pink rows in sub-table 3 are the dams followed by the rows of their respective offspring.

Tan rows are champagne.

Blue rows are non-champagne.

Markers of recombinants are in red.

Markers associated with champagne from the sire are tan.

Non-champagne markers from sire are in blue.

-- designates no data for that marker

CH denotes champagne color

Ch denotes non-champagne
