# Supplementary material for: Missense Mutation in Exon 2 of SLC36A1 Responsible for Champagne Dilution in Horses
Source: PLoS Genet. 2008 Sep 19;4(9):e1000195. doi: 10.1371/journal.pgen.1000195 (PMC2535566; doi:10.1371/journal.pgen.1000195)
Supplement: Table S2 — Sequence Variants Detected in SPARC, SLC36A1, and SLC36A2. (0.14 MB DOC) [file pgen.1000195.s003.doc]

| **Sequence Variations Detected** | | | | | | | | |
| --- | --- | --- | --- | --- | --- | --- | --- | --- |
| **Gene** | **Location** | **Loc. Size** | **Base Position** (within location) | **Variation** | **Type** (Exons) | **5' flank** | **3'flank** | **residues** |
| ***SPARC*** | Intron 1-2 | 1457 | 1353 | A>G |  | ATG | GAATCTCCAG |  |
|  |  |  | 1365 | C>T |  | GAATCTCCAG | GAGCTGCTCTCC |  |
|  |  |  |  |  |  |  |  |  |
| ***SPARC*** | Exon 2 | 63 | 60 | C>T | synonomous | GGCTGAGGTGTC | GAGGTACGTGGG | Cys>Cys |
|  |  |  |  |  |  |  |  |  |
| ***SPARC*** | Intron 2-3 | 634 | 157 | G>A |  | TGCGTATTCCCA | T |  |
|  |  |  | 561 | C>T |  | AAGCCTCCCC | CAGTCTTCACCC |  |
|  |  |  |  |  |  |  |  |  |
| ***SPARC*** | Intron 3-4 | 1463 | 87 | C>T |  | TTCTGTGAGGTT | CCTCCCAAGGGA |  |
|  |  |  | 1397 | G>A |  | AAGGATTGGTCT | GATTGGGTTGGG |  |
|  |  |  |  |  |  |  |  |  |
| ***SPARC*** | Exon 4 | 122 | 69 | T>C | missense | CAGTCCCATGTG | GTGTGCCAGGAC | Cys>Arg |
|  |  |  |  |  |  |  |  |  |
| ***SPARC*** | Intron 4-5 | 1845 | 1827 | C>T |  | AAAGGTGCGAAA | CCTGTGCTTTGC |  |
|  |  |  |  |  |  |  |  |  |
| ***SPARC*** | Intron 5-6 | 2222 | 19 | G>C |  | CTTTTCCTTGGGC | CTTCTGGGCTTC |  |
|  |  |  | 49 | C>A |  | CGAGGAGAGACC | CTGAACTGACGC |  |
|  |  |  | 78 | A>G |  | GCTGGCTGTGCC | CCCCC |  |
|  |  |  | 2181 | A>G |  | ACCTTGGGCGGG | AGAGTCAGGGTC |  |
|  |  |  |  |  |  |  |  |  |
| ***SPARC*** | Intron 6-7 | 676 | 645 | T>C |  | AGTCTCCCTGGA | GCTTTGTCACAG |  |
|  |  |  |  |  |  |  |  |  |
| ***SPARC*** | Exon 7 | 149 | 51 | A>G | synonomous | TGGAGACCACCC | GTGGAGCTGCTG | Ser>Gly |
|  |  |  |  |  |  |  |  |  |
| ***SPARC*** | Intron 7-8 | 2113 | 95 | T>G |  | GGTAGGCTCACT | GATATGGTCAGA |  |
|  |  |  |  |  |  |  |  |  |
|  |  |  |  |  |  |  |  |  |
| ***SLC36A1*** | Exon 2 | 91 | 76 | C>G | missense | GCAACATTGGCA | AGGACTCCTGGG | Thr >Arg |
|  |  |  |  |  |  |  |  |  |
|  |  |  |  |  |  |  |  |  |
| ***SLC36A2*** | Upstream |  | - 89 from Start | C> | deletion | GCTGCTGAGA | GGGGTTTGCACT |  |
|  |  |  |  |  |  |  |  |  |
| ***SLC36A2*** | Intron 2-3 | 564 | 9 | G>C |  | CTGGTAAGAAGG | GCTGCCGCAGGT |  |
|  |  |  |  |  |  |  |  |  |
| ***SLC36A2*** | Exon 6 | 219 | 45 | C>T | synonomous | TTACAA | GAGACAGTGATT | Tyr>Tyr |
|  |  |  |  |  |  |  |  |  |
| ***SLC36A2*** | Intron 6-7 | 1385 | 1339 | T>C |  | ATTCAGGTCTAC | GTCAGAATACTC |  |
|  |  |  | 1308 | T>A |  | TAAAGGAGCTCA | ACATGCTTCGAG |  |
|  |  |  |  |  |  |  |  |  |
| ***SLC36A2*** | Exon 9 | 170 | 94 | C>T | synonomous | CCTCTACGTCTT | GGCATCCTGTGC | Trp>Trp |
|  |  |  | 155 | G>A | synonomous | ATCGTCCCCTTC | CTGTCTCCCGCGC | Ser>Ser |
|  |  |  |  |  |  |  |  |  |
| ***SLC36A2*** | Intron 9-10 | 2456 | 23 | T>G |  | TCAGCCAGATGG | GAAGGATGGT |  |
|  |  |  | 33 | T>G |  | GAAGGATGG | ? |  |
|  |  |  |  |  |  |  |  |  |
| ***SLC36A2*** | Exon 10 | 354 | 50 | T>G | missense | CCTCTCCCTGGT | GGCTCCATGAGC | Trp>Gly |
|  |  |  | 64 | G>C | missense | GCTCCATGAGCA | CAGCGCCCTGGC | Cys>Ser |
|  |  |  | 67 | G>C | missense | CCATGAGCACCA | CGCCCTGGCCCT | Cys>Ser |
|  |  |  | 227 | A>G | synonomous | TCTGATCCAGCC | ACAGACCATCTC | Ser>Ser |
|  |  |  | 265 | C>T | missense | CCACCATTTTCA | TCAGTGAGAATG | Ser>Phe |
|  |  |  | 269 | G>C | missense | GATTTTCACTCA | TGAGAATGGTGC | Cys>Ser |
|  |  |  |  |  |  |  |  |  |

Supplemental Table 2:

Exon 2 variation in *SLC36A1* was the only variation detected in that gene.

Flanking sequences provided are from sequencing traces to assist any wishing to locate regions for these SNPs by BLAST search.
