# Supplementary material for: Missense Mutation in Exon 2 of SLC36A1 Responsible for Champagne Dilution in Horses
Source: PLoS Genet. 2008 Sep 19;4(9):e1000195. doi: 10.1371/journal.pgen.1000195 (PMC2535566; doi:10.1371/journal.pgen.1000195)
Supplement: Table S3 — Microsatellite Markers used For Genome Scan. (0.20 MB DOC) [file pgen.1000195.s004.doc]

| Marker | Chromosome | Alleles | Size | Annealing Temp | Optimal concentration (uM) |
| --- | --- | --- | --- | --- | --- |
|  |  |  |  |  |  |
| AHT21 | 01 | 8 | 199-215 | 58 | 0.09 |
| COR100 | 01 | 7 | 212-230 | 56 | 0.15 |
| HMS15 | 01 | 11 | 207-245 | 56 | 0.6 |
| VIAS-H34 | 01 | 7 | 144-160 | 58 | 0.7 |
| ASB8 | 01 | 8 | 138-164 | 58 | 0.08 |
| LEX020 | 01 | 7 | 198-222 | 58 | 0.12 |
| ASB41 | 01 | 6 | 156-168 | 58 | 0.3 |
| NVHEQ100 | 01 | 7 | 197-217 | 58 | 0.1 |
| ASB18 | 02 | 12 | 196-213 | 58 | 0.15 |
| A-14 | 02 | 10 | 220-248 | 58 | 0.8 |
| COR065 | 02 | 9 | 280-292 | 58 | 0.3 |
| ASB17 | 02 | 17 | 93-125 | 58 | 0.1 |
| UM007 | 02 | 18 | 122-176 | 58 | 0.05 |
| COR033 | 03 | 9 | 222-254 | 58 | 0.2 |
| ASB23 | 03 | 7 | 187-213 | 58 | 0.1 |
| UCDEQ437 | 03 | 8 | 167-193 | 58 | 0.1 |
| SGCV23 | 04 | 8 | 221-233 | 56 | 0.6 |
| ASB22 | 04 | 9 | 155-177 | 58 | 0.1 |
| COR089 | 04 | 10 | 282-304 | 58 | 0.1 |
| LEX004 | 05 | 6 | 282-300 | 58 | 0.15 |
| LEX069 | 05 | 7 | 248-262 | 56 | 0.6 |
| LEX034 | 05 | 6 | 252-262 | 58 | 0.05 |
| TKY28 | 06 | 7 | 280-364 | 58 | 0.15 |
| COR088 | 06 | 7 | 283-297 | 58 | 0.4 |
| COR070 | 06 | 12 | 279-307 | 58 | 0.2 |
| NVHEQ82 | 06 | 6 | 133-147 | 58 | 0.01 |
| VIAS-H7 | 07 | 12 | 116-146 | 58 | 0.3 |
| COR004 | 07 | 6 | 297-319 | 58 | 0.5 |
| COR003 | 08 | 8 | 195-215 | 58 | 0.15 |
| COR056 | 08 | 10 | 194-220 | 58 | 0.2 |
| LEX023 | 08 | 12 | 233-257 | 58 | 0.15 |
| ASB14 | 08 | 9 | 118-136 | 58 | 0.05 |
| HTG8 | 09 | 7 | 185-197 | 56 | 0.6 |
| ASB4 | 09 | 6 | 128-140 | 58 | 0.7 |
| COR008 | 09 | 12 | 251-277 | 58 | 0.12 |
| UM037 | 09 | 7 | 108-124 | 58 | 0.2 |
| ASB6 | 10 | 8 | 185-212 | 58 | 0.6 |
| COR048 | 10 | 10 | 178-186 | 58 | 0.3 |
| ASB9 | 10 | 9 | 67-113 | 58 | 0.3 |
| NVHEQ18 | 10 | 15 | 119-161 | 58 | 0.06 |
| COR020 | 10 | 7 | 162-176 | 58 | 0.2 |
| SGCV24 | 11 | 10 | 125-141 | 56 | 0.5 |
| SGCV13 | 11 | 5 | 169-179 | 58 | 0.7 |
| LEX068 | 11 | 7 | 162-174 | 58 | 0.12 |
| SGCV8 | 12 | 8 | 126-143 | 58 | 0.2 |
| SGCV10 | 12 | 6 | 179-187 | 56 | 0.6 |
| AHT17 | 12 | 11 | 123-147 | 58 | 0.1 |
| COR058 | 12 | 12 | 218-244 | 58 | 0.1 |
| ASB37 | 13 | 6 | 132-146 | 58 | 0.04 |
| COR069 | 13 | 8 | 273-287 | 58 | 0.13 |
| VHL047 | 13 | 4 | 134-150 | 58 | 0.05 |
| COR002 | 14 | 5 | 235-243 | 58 | 0.05 |
| UM010 | 14 | 7 | 112-126 | 58 | 0.05 |
| VHL209 | 14 | 6 | 91-105 | 58 | 0.2 |
| AHT16 | 15 | 7 | 130-153 | 58 | 0.2 |
| COR014 | 15 | 12 | 149-164 | 58 | 0.04 |
| B-8 | 15 | 8 | 88-110 | 56 | 0.1 |
| COR075 | 15 | 9 | 202-220 | 58 | 0.07 |
| HMS20 | 16 | 8 | 116-140 | 58 | 0.25 |
| L15.2 | 16 | 9 | 147-165 | 58 | 0.07 |
| LEX056 | 16 | 7 | 218-234 | 58 | 0.08 |
| I-18 | 16 | 9 | 93-119 | 58 | 0.05 |
| COR007 | 17 | 9 | 163-177 | 58 | 0.04 |
| LEX055 | 17 | 7 | 216-232 | 58 | 0.15 |
| NVHEQ79 | 17 | 7 | 175-197 | 58 | 0.08 |
| TKY19 | 18 | 9 | 147-173 | 56 | 0.04 |
| COR096 | 18 | 8 | 315-329 | 58 | 0.2 |
| LEX054 | 18 | 10 | 170-190 | 58 | 0.08 |
| COR092 | 19 | 6 | 191-203 | 58 | 0.1 |
| LEX036 | 19 | 8 | 148-170 | 58 | 0.05 |
| LEX073 | 19 | 11 | 249-277 | 58 | 0.15 |
| LEX052 | 20 | 7 | 208-214 | 58 | 0.05 |
| LEX071 | 20 | 7 | 192-211 | 58 | 0.12 |
| HMS42 | 20 | 0 | 132-140 | 58 | 0.07 |
| UM011 | 20 | 11 | 167-187 | 58 | 0.1 |
| COR073 | 21 | 8 | 187-205 | 58 | 0.1 |
| LEX037 | 21 | 4 | 196-202 | 56 | 0.07 |
| SGCV16 | 21 | 5 | 154-194 | 58 | 0.12 |
| HMS47 | 22 | 7 | 203-215 | 58 | 0.17 |
| HTG21 | 22 | 7 | 131-143 | 58 | 0.08 |
| COR016 | 22 | 7 | 184-203 | 58 | 0.12 |
| COR055 | 23 | 9 | 240-270 | 58 | 0.1 |
| LEX074 | 24 | 10 | 155-175 | 58 | 0.15 |
| COR024 | 24 | 6 | 214-226 | 58 | 0.05 |
| COR061 | 24 | 11 | 197-227 | 58 | 0.1 |
| COR018 | 25 | 7 | 251-283 | 58 | 0.1 |
| NVHEQ70 | 26 | 7 | 192-208 | 58 | 0.08 |
| COR071 | 26 | 8 | 188-210 | 58 | 0.05 |
| A-17 | 26 | 8 | 102-118 | 58 | 0.15 |
| COR017 | 27 | 12 | 241-267 | 58 | 0.15 |
| COR031 | 27 | 7 | 210-224 | 58 | 0.08 |
| COR040 | 27 | 8 | 282-300 | 58 | 0.25 |
| UCDEQ425 | 28 | 8 | 236-250 | 58 | 0.1 |
| COR027 | 29 | 7 | 231-255 | 56 | 0.12 |
| COR082 | 29 | 7 | 199-233 | 58 | 0.4 |
| L12.2 | 29 | 10 | 136-156 | 58 | 0.05 |
| LEX025 | 30 | 7 | 152-168 | 58 | 0.04 |
| LEX075 | 30 | 8 | 144-164 | 58 | 0.04 |
| COR038 | 31 | 4 | 210-214 | 58 | 0.1 |
| AHT33 | 31 | 8 | 151-167 | 58 | 0.15 |
| UM038 | X | 7 | 120-144 | 58 | 0.2 |
| LEX022 | X | 7 | 110-124 | 58 | 0.12 |

Table S3: Microsattelite list used for genome scan.
