# Supplementary material for: Missense Mutation in Exon 2 of SLC36A1 Responsible for Champagne Dilution in Horses
Source: PLoS Genet. 2008 Sep 19;4(9):e1000195. doi: 10.1371/journal.pgen.1000195 (PMC2535566; doi:10.1371/journal.pgen.1000195)
Supplement: Table S4 — Sequencing and RT-PCR Primers. (0.07 MB DOC) [file pgen.1000195.s005.doc]

| **Table S4 Primers Used**  **Part 1: Primer sequences for the sequencing genomic exons of horse *SLC36A1*** | | | | | | | | | |
| --- | --- | --- | --- | --- | --- | --- | --- | --- | --- |
| Exon # | | Forward Primer (5’-3’) | | | | Reverse primer (5’-3’) | | Product Size | |
|  | |  | | | |  | |  | |
| 1 | | AAGCGCCTGTCTGTCTCTTC | | | | CTCTTCCTCAGCACCAGCTT | | 709 | |
| 2 | | CAGAGCCTAAGCCCAGTGTC | | | | GGAGGACTGTGTGGAAATGG | | 711 | |
| 3 | | ATCCCAGGAGCCTCTGTTCT | | | | GAAGCGTTAAGCCAACAGGA | | 696 | |
| 4 | | CAGCTGCTCAGCATCACAG | | | | CAAAGCAGGAACAGCCCTTA | | 699 | |
| 5 | | CTCCTGCCATTCCAGTCTTG | | | | AAGGCTCAGTGTGTGAACGA | | 702 | |
| 6 | | AACCCAGCTCAGACAGTTGG | | | | CCAGAGACCTTTGGCAATGT | | 685 | |
| 7 | | TTTGTGCATGCTCCAACATT | | | | GTCAAGTCCTCGTGCAAGGT | | 697 | |
| 8 | | CTTATCGCAGGAGGCAGAAC | | | | GTCCCACATGGGTAACAAGG | | 700 | |
| 9 | | TATCGTGGAGCTGGTTGTGT | | | | TTCTCTGCACCATCTGGACA | | 699 | |
| 10 | | CTTGCAGTGAGAGACAGGTTATTC | | | | TGGGGAACATACAGCGGT | | 509 | |
| **Part2: Primer sequences for the sequencing genomic exons of horse *SLC36A2*** | | | | | | | | | |
| Exon # | Forward Primer (5’-3’) | | | | Reverse primer (5’-3’) | | Product Size | | |
|  |  | | | |  | |  | | |
| 1 | CAGAGCCTGCTAAGGCACAC | | | | TTTTCCTCAGCTGCACAATG | | 403 | | |
| 2 | GACTGTGAGAAGGCCAGGAG | | | | GCTTGTGGAGGCCACTCTAA | | 407 | | |
| 3 | AAAAGGGCTTGGAAACCAGT | | | | AGCAGAACCTCGCCTTAGGT | | 392 | | |
| 4 | CTGCCACAGTGTTCTTTCCA | | | | CACCCTGTTGCTGGAGGTAT | | 403 | | |
| 5 | GGGACAGAAATGGAAACGAC | | | | CCTTGAGGACAGGTCCAAGA | | 402 | | |
| 6 | CACCCTAACTCGCTGAGACC | | | | ATAAAAGGCTCTGCCCACTG | | 401 | | |
| 7 | TCCTCTGGCTCTTTGGTTGT | | | | AGGCCAGACGTTGCTTTCT | | 404 | | |
| 8 | CCTTTCACCCATCAATGGAC | | | | TAGCCTTGAGTCCCCATCAC | | 400 | | |
| 9 | CTGCTCTGACTCCCTCTTGG | | | | CTGGTTCCTGACCATCCTTC | | 398 | | |
| 10 | GTCTAAGCCTGGGATGATGC | | | | TCGTAGCTGGTGAATGCTTG | | 456 | | |
| **Part 3: Primer sequences for reverse transcription and sequencing of partial cDNAs from mRNA** | | | | | | | | | |
| **Gene** | | | **Forward Primer (5’-3’)** | **Reverse primer (5’-3’)** | | | **Product**  **(bp)** | | **Exons**  **Included** |
|  | | |  |  | | |  | |  |
| *SLC36A1* | | | ACCAGCGGTTTGGGGAAA | ACGGCCACGATGCCCATCA | | | 169 | | 1-3 |
| *SLC36A2 (outer long)* | | | aagcgtcatgcccgtgacaaagagtgcg | gtggcagaagcgctgggcacacctg | | | 348 | | 1-3 |
| *SLC36A2 (outer short)* | | | GCCCGTGACAAAGAGTGCG | AAGCGCTGGGCACACCTG | | | 334 | | 1-3 |
| *SLC36A2 (nested)* | | | ACCTCAAACTGGACCTCAGG | ATGCCTGCGTTCCTCACA | | | 208 | | 2-3 |
| *SLC36A3* | | | TTGGAAGGGACTACAACAGTGAG | GGTTTCGAGGCTGTACATCATG | | | 350 | | 1-4 |

Table S4: Primers used for Sequencing and RT-PCR
